# Protein Binding Characteristics of the Principal Green Tea Catechins: A QCM Study Comparing Crude Extract to Pure EGCG

**DOI:** 10.1155/2019/6154170

**Published:** 2019-10-30

**Authors:** Elsadig E. Ali, Mohamed O. Elmakki, Miranda L. Gavette, Brian J. Doyle, Shannon J. Timpe

**Affiliations:** ^1^Department of Mechanical Engineering, Bradley University, Peoria, IL, USA; ^2^Departments of Biology and Biochemistry, Alma College, Alma, MI, USA

## Abstract

Label-free detection methods such as the quartz crystal microbalance (QCM) are well suited to the analysis of molecular interactions in complex mixtures such as crude botanical extracts. In the present study, the binding characteristics of epigallocatechin gallate (EGCG) and crude green tea extract solutions to bovine serum albumin (BSA) have been investigated. The adsorbed mass levels onto BSA-functionalized surfaces were measured at various solution concentrations. Langmuir and Freundlich isotherms were used to model the adsorption data. The Langmuir isotherm better described the adsorption behavior with correlations of 0.68 and 0.70 for the EGCG and the crude extract solutions, respectively. The better fit of the Langmuir model indicates that adsorption occurs homogeneously and that aggregation is negligible. The mass saturation is estimated to be 58% higher for the crude green tea solution as compared to the pure EGCG solution (7.9 ng/cm^2^ for green tea and 5 ng/cm^2^ for EGCG). The increased adsorption for the crude extract indicates that the additional tea chemical constituents are binding to alternate sites on the protein molecule and that competitive binding is a nondominant effect. However, a reduced adsorption rate for the crude extract was also observed, indicating some presence of competitive mechanisms. The results demonstrate the utility of the QCM for the analysis of protein binding in crude mixtures as well as pure compounds.

## 1. Introduction

Natural products are an important source of therapeutic agents with more than 75% of the modern drugs used to treat infectious diseases and 60% of the drugs used to treat cancer comprised of either natural products or synthetic molecules inspired by the pharmacology of natural products [1], such as Etoposide, Teniposide, Paclitaxel (Taxol), morphine, digitoxin, quinine, and atropine [2–5]. Despite advances in synthetic chemistry, investigation of complex and diverse natural products as potential pharmaceuticals remains an active area of research.

**Figure 1:**
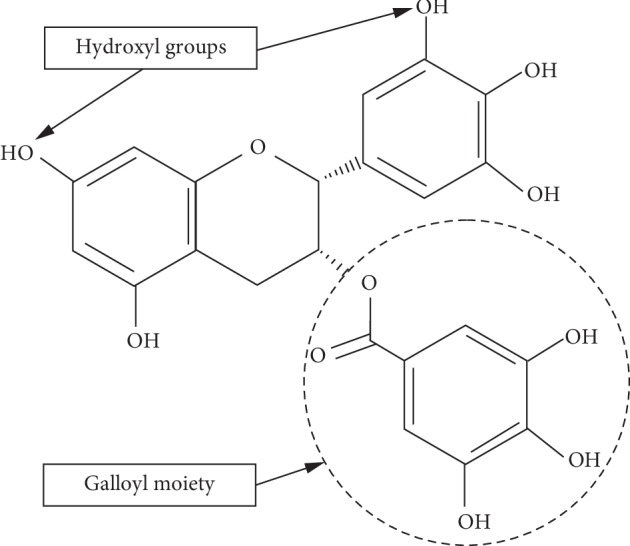
Structure of (−)-epigalocatechin gallate (EGCG). The hydroxyl and galloy groups are instrumental in the binding of EGCG to bovine serum albumin.

Tea (*Camellia sinensis)* is widely consumed throughout the world and is the most popular beverage after water [6]. Tea polyphenols are mainly composed of catechins, flavonoids, and phenolic acid. The catechins comprise more than 80% of the content, and the biological activity of tea is primarily attributed to these catechins [7]. The major catechins of green tea are epicatechin (EC), (−)-epigalocatechin (EGC), (−)-epicatechin gallate (ECg), and epigallocatechin gallate (EGCG).  Figure 1 shows the chemical structure of EGCG, the principal catechin examined in the current study and the compound thought to be responsible for the health benefits of green tea [8, 9]. The distinguishing structural characteristic among these catechins lies in the presence or absence of the galloyl moiety and the hydroxyl groups on the benzene ring [10]. Minoda et al. [11] showed that the interaction of EGCG and ECg with HSA was 100 times stronger than the interaction of the catechins lacking the galloyl moiety. Tea polyphenols have various pharmacological properties, for example, counteracting harmful oxidant radicals associated with multiple diseases such as heart disease and cancer [12, 13], treatment of benign prostate hyperplasia [14], and lowering risk of biliary tract cancer (BTC) [15]. It should be noted that green tea catechins readily oxidize in aqueous solutions at neutral to alkaline pH, which reduces their bioactivity. Catechins bind to serum albumin enabling their distribution to the tissues. Interestingly, it has been suggested that catechins are stabilized when bound to serum albumin due to the antioxidant effect of free sulfhydryl groups in the protein [16].

Albumin is the most abundant plasma protein in humans and is an attractive model protein to study drug binding because it acts as a molecular transport to multiple ligand types [17]. Bovine serum albumin (BSA) is a globular protein with three major domains (І, II, and III), each of which is comprised of two helical subdomains A and B [18]. Deep hydrophobic pockets with positively charged entrances are located at similar positions in subdomains IIA and IIIA, and these pockets are thought to correspond to the binding sites for various compounds [19]. For example, the binding site in the domain IIIA is known to be able to bind to the drugs digitoxin, aspirin, and ibuprofen. Aspirin and iodinated aspirin derivatives show nearly equal distributions between subdomains IIA and IIIA, while warfarin occupies a single site in IIA [20].

Detection and quantification of molecular interactions is critical in screening, characterizing, and discovering new drugs. The major techniques of detection can be classified as either label-based or label-free. While label-based detection is extensively used due to the reagent and instrument availability, labeling strategies can alter the natural activity of the molecules under study [21]. Conversely, label-free detection methods depend on direct property measurements of the molecule under study. Label-free techniques are particularly advantageous in the development of arrays for characterizing multiple molecular interactions [22]. The quartz crystal microbalance (QCM) is an instrument that enables label-free detection of molecular interactions through the measurement of changes in the resonant frequency of a piezoelectric crystal due to adsorbed mass.

In the current study, a QCM is used to study the protein binding characteristics of a solution of pure EGCG and a crude green tea extract and compare the adsorption behavior and magnitude of both solutions to the same protein (i.e., BSA). Results are interpreted in light of the principal binding mechanisms, the efficiencies of those mechanisms, and the complex interactions present in crude botanical extracts.

## 2. Experimental

### 2.1. Material, Reagents, and Solutions

Bovine serum albumin and epigallocatechin gallate were purchased from Sigma-Aldrich (St. Louis, MO, USA) and used without further purification. The green tea extract was prepared from a commercial extract, EGCG Green Tea Extract (NOW Foods, Bloomingdale, IL, USA). The contents of 50 capsules were dissolved in 500 mL of deionized water and filtered with Fisher Scientific P4 medium-fine porosity filter paper to remove excipients (silica and magnesium stearate), and water was evaporated *in vacuo*.

The BSA solutions were prepared to a concentration of 10^−5^ mol/L by dissolving the appropriate mass in phosphate buffered saline. The buffer was prepared by dissolving 80 g/L of NaCl, 2 g/L of KCl, 14.4 g/L of Na_2_HPO_4_, and 0.244 g/L of KH_2_PO_4_ in deionized water; then the buffer pH level was adjusted to 4.9 using HCl. The EGCG solutions were prepared at concentrations ranging from 10^−6^ to 10^−3^ mol/L by dissolving the required masses in sodium acetate buffer. This buffer consisted of 10.5% of glacial acetic acid, 39.5% of sodium acetate, and 50% deionized water, and the buffer's pH was also adjusted to 4.9. Although this pH level does not represent the physiological human body pH (7.4), previous studies have shown that a pH of 4.9 is optimal for the investigation of catechin-BSA interactions because catechins are more stable at acidic pH, and BSA retains its native conformation at pH 4.9 [23]. The crude green tea extract solution was prepared by dissolving the appropriate solid masses in sodium acetate buffer at concentrations ranging from 2.0 × 10^−6^ to 2.0 × 10^−3^ mol/L. With the 47% EGCG content confirmed with high-performance liquid chromatography, these solid concentrations for the crude extract correspond to EGCG concentrations ranging from approximately 10^−6^ to 10^−3^ mol/L, thus matching the EGCG content range of the pure EGCG solution.

### 2.2. QCM Measurement Techniques

A quartz crystal microbalance (QCM200, Stanford Research Systems, Sunnyvale, CA, USA) was used in order to compare the protein binding characteristics of pure EGCG solution with that of a crude green tea extract solution. The sensor consists of a 1.37 cm^2^ polished gold electrode on a 2.54 cm diameter AT-cut quartz crystal (O100RX3, Stanford Research Systems, Sunnyvale, CA, USA). Prior to use, crystals were cleaned for 2.0 min in Piranha solution in order to remove organic residue. Piranha solution was prepared at a 3 : 1 ratio of sulfuric acid to 30% hydrogen peroxide. After cleaning, the crystals were rinsed in deionized water and dried using compressed air. A 150 *μ*L flow cell (O100FC, Stanford Research System, Sunnyvale, CA, USA) was used to introduce the solutions to the crystal surface. A syringe pump (NE-300, New Era Pump Systems Inc., Farmingdale, NY, USA) was used to introduce the solutions to the crystal via chemical resistant tubing. The syringe pump was operated at a constant flow rate of 0.1 mL/min. No external temperature control was used during this study. However, all solutions were introduced to the flow cell at room temperature, and no significant ambient temperature changes were observed during the experiments.

### 2.3. Adsorption Measurements

In order to create a protein-functionalized surface, BSA was immobilized onto the gold electrode of the crystal, resulting in a saturated monolayer [24]. The protein binds to the bare gold via at least one thiol originating from its 17 disulfide bonds [25, 26]. In previous work, it was found that immobilizing the BSA directly to the gold surface was adequate and gave similar mass density compared with functionalizing the surface with a linker prior to introducing BSA [24, 27]. The mass adsorption characteristics of EGCG and crude green tea solutions were measured by first introducing sodium acetate buffer in order to establish a steady state baseline resonant frequency; the change in mass associated with the adsorption of the green tea constituents results in a corresponding change in the crystal resonant frequency. The change in frequency can be used to estimate the adsorbed mass via the Sauerbrey equation [28]

(1) $$\begin{array}{c} \Delta f=-C_{f}\Delta m, \end{array}$$

where *C*_*f*_ is the sensitivity factor (56.6 Hz-cm^2^·*μ*g^−1^) for a 5 MHz AT-cut quartz crystal at room temperature. Note that the Sauerbrey equation is applicable only to rigid, uniform thin films [29]. However, it fails to accurately characterize the adsorption of viscoelastic thin film such as the type examined in the current study. In addition, it should be noted that BSA changes its conformation upon binding of ligands [20, 30, 31], which will cause a change in the resonant frequency of the QCM. In order to account for viscoelasticity and BSA conformation changes, a new factor was introduced to the Sauerbrey equation

(2) $$\begin{array}{c} \Delta f=-C_{v}C_{f}\Delta m, \end{array}$$

where the viscoelastic factor *C*_*v*_ can be estimated by comparing the experimental data to the theoretical estimations. This provides a simple means for making quantitative comparisons and circumventing the inaccuracies inherent in the assumptions of the Sauerbrey equation.

**Figure 2:**
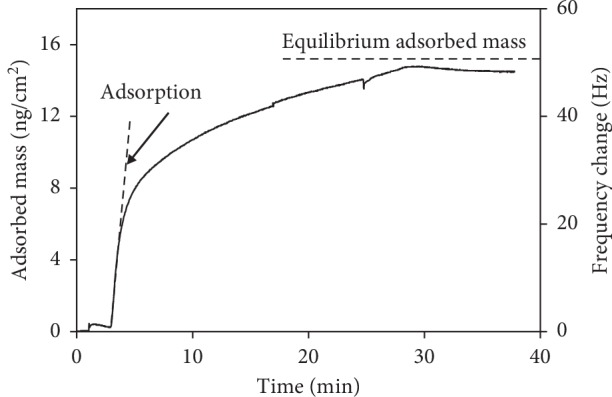
Characteristic adsorption curve showing the change in resonant frequency and the estimated adsorbed mass as a function of time.

Figure 2 shows a characteristic plot of the change in resonant frequency (and estimated adsorbed mass per unit area) as a function of time. This particular plot represents an experiment involving binding of EGCG from a 10^−3^ mol/L concentration solution onto the BSA-functionalized surface. This figure shows the adsorption rate, defined as the initial slope of the adsorption curve, and the equilibrium adsorbed mass, in this case, achieved after approximately 30 minutes of exposure to the test solution. It should be noted that ambient temperature changes will affect the frequency magnitude [32]. However, since the adsorbed mass is based on the difference between the two steady frequency lines, and no significant temperature changes occurred during the experiments, the temperature effect on the results is expected to be minimal. No external temperature control was utilized during this study.

## 3. Results and Discussions

### 3.1. High-Performance Liquid Chromatography

High-performance liquid chromatography was used to determine the EGCG content of the experimental samples. A standard curve was first obtained by analyzing samples of the pure EGCG solution at a range of concentrations from 0.125 to 2.0 mg/mL. Linear regression analysis resulted in an equation that was then used to determine that the EGCG content of the green tea extract was 47% (±3.4%). This result is in agreement with the 50% EGCG content claimed by manufacturer.

### 3.2. Green Tea and EGCG Binding

Prior to each green tea or EGCG experiment, BSA was immobilized onto the gold electrode of the QCM crystal to create a saturated monolayer. The average frequency change between the baseline and BSA saturation was 21 Hz, corresponding to 375 ng/cm^2^, which is consistent with previous studies [24]. Furthermore, BSA mass saturation can be estimated by modeling each BSA molecule as a triangular prismatic shell with dimensions of 8.4 nm × 8.4 nm × 8.4 nm × 3.2 nm [33]. Using this model, and knowing the QCM crystal area, the amount of BSA can be estimated to be 4.5 × 10^12^ molecules/crystal, or 363 ng/cm^2^. Thus, it can be said with high confidence that BSA monolayer saturation was achieved.

EGCG is typically taken along with other chemical compounds in the form of an extract, i.e., a tea or a dietary supplement pill; therefore, the influence of other green tea constituents on EGCG pharmacokinetics should be understood. In the present study, a QCM was used to investigate the BSA-binding characteristics of a pure EGCG solution and an EGCG-containing crude botanical extract (green tea) solution. The results suggest that other green tea constituents do not interfere with distribution of EGCG through competition for binding sites on serum albumin.

**Figure 3:**
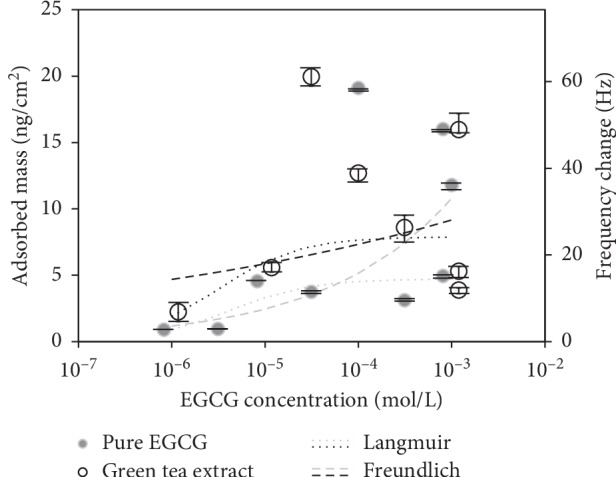
Adsorbed mass from EGCG and green tea solutions as a function of concentration. Each data point represents a complete experiment on a clean, BSA-functionalized crystal. The Langmuir and Freundlich isotherm regression curves are also shown.

Figure 3 shows the adsorbed mass for EGCG and green tea solutions as a function of concentration. It should be noted that green tea extract data points are placed according to EGCG concentration (47% of total solid concentration). Each data point represents a single adsorption experiment with a clean crystal. A wide range of concentrations were tested, and replicates were performed at selected concentrations in order to determine the behavioral isotherm while simultaneously allowing for an estimation of the statistical variation through a statistical analysis of the coefficient of determination. The adsorption data was analyzed using the two general equilibrium adsorption models of Langmuir and Freundlich and these curve fits are also shown in Figure 3. The Langmuir isotherm is used extensively to describe adsorption processes in which there are a specific number of available binding sites in the adsorbent. These sites are filled until the molecule is fully saturated and further adsorption is inhibited. The Langmuir model also assumes that when a site is occupied by a molecule, no other molecule can bind to that site [34]. The Langmuir equation is expressed as follows:

(3) $$\begin{array}{c} \Gamma =\Gamma_{\max}\frac{\alpha c}{1+\alpha c}, \end{array}$$

where *Γ* is the adsorbed mass, *Γ*_max_ is the amount of adsorbed mass at saturation, *α* is the Langmuir adsorption constant, and *c* is the solution concentration. Multiple linearizations of the basic Langmuir equation were used to determine the fitting constants with the best fit obtained using the Lineweaver–Burk linearization:

(4) $$\begin{array}{c} \frac{1}{\Gamma }=\frac{1}{\Gamma_{\max}}+\frac{1}{\alpha \Gamma_{\max}}\cdot \frac{1}{c}. \end{array}$$

The resulting constants for the adsorption from the pure EGCG solution and that from the green tea solution are shown in Table 1.

The empirical Freundlich equation was also used to fit the current adsorption data. This model assumes adsorption onto a heterogeneous surface and is expressed as follows:

(5) $$\begin{array}{c} \Gamma =k_{f}c^{1/n}, \end{array}$$

where *k*_*f*_ is a constant related to the adsorption capacity, and *n* is a constant related to the adsorption intensity [35]. The best fit Freundlich constants are also shown in Table 1.

The results show that the adsorption behaviors for both EGCG and the crude extract are best described by the Langmuir model, as indicated by the higher determination coefficients. One of the characteristics of Langmuir isotherm behavior is reaching mass saturation [33]. This saturation can be seen as a horizontal line in the frequency shift versus concentration plot. In this study, saturation was reached at solution concentrations of around 3.0 × 10^−5^ mol/L for both green tea and pure EGCG solutions. Higher concentrations were tested to confirm that saturation had been achieved, and a concentration range from 10^−6^ to 10^−3^ was found to be sufficient to characterize the molecular interaction in light of the isotherms used in this study. The better fit of data to the Langmuir isotherm suggests that the catechin adsorption onto BSA occurs homogeneously and that there is limited multilayer formation or molecular aggregation. This is expected as specific interaction is facilitated via the formation of hydrogen bonds between the phenolic groups in the tea polyphenols and carbonyl groups in the proline-rich BSA molecule [36]. Furthermore, the experimental replicates performed were used to calculate the lack of fit and pure error *P* values for the linearization model. From Tables 2 and 3, the *P* values for EGCG and green tea were found to be 0.28 and 0.79, respectively. Indicating that, with 95% confidence, there is no evidence for lack of fit of the Lineweaver–Burk Langmuir linearization model. This statically analysis was conducted to raise the confidence of the results shown in this study and proving that results can be used for further analysis. In summary, green tea and EGCG adsorption results showed that both the crude extract and pure EGCG followed the same trend, meaning there were no major changes to the adsorption mode of green tea and EGCG, and the results can be used to further analyze and compare the binding of the two solutions to BSA.

**Figure 4:**
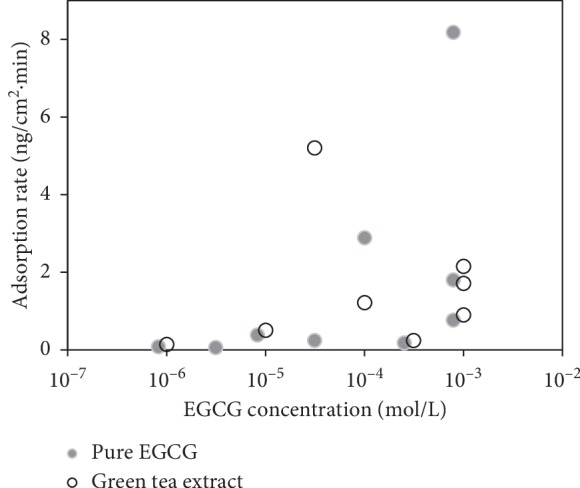
Adsorption rates from EGCG and green tea solutions as a function of concentration. Each data point represents a complete experiment measurement on a clean and BSA-functionalized crystal. Data points are defined by the slope of mass vs. time curve at the beginning of the adsorption process (see Figure 2).

BSA is composed of three structural domains (I, II, and III), each of which is formed by two subdomains, A and B. However, subdomains IIA and IIIA are the principle binding sites [37]. Hence, to achieve full saturatation, as assumed by the Langmuir model, two molecules of EGCG will bind at these two subdomains. Taking this into account, the mass saturation of EGCG can be estimated by recalling that there are 4.5 × 10^12^ BSA molecules/crystal. Two molecules of EGCG will bind to each protein molecule resulting in a total 6.6 × 10^12^ EGCG molecules/cm^2^. Using the molar mass of EGCG (458.4 g/mol), the homogenous mass saturation is estimated to be 5.0 ng/cm^2^. This theoretical mass saturation can be compared to the mass saturation estimated using the Sauerbrey equation (1) in order to determine the viscoelastic factor *C*_*v*_. Using this methodology, a value of 53.6 was obtained for *C*_*v*_ and used to scale the secondary *y*-axis in Figures 2–4. This large disparity between the rigid film estimation and the viscous film estimation is expected as BSA is a relatively large globular protein [38].

A higher BSA-binding mass was observed for the crude green tea extract (7.9 ng/cm^2^) than for pure EGCG (5.0 ng/cm^2^) (Figure 3), which is consistent with the fact that green tea contains multiple polyphenolic compounds that bind to BSA, including EC, EGC, ECg, and EGCG. Results showed that competitive binding is not dominant since EGCG has the highest molar mass of all the green tea catechins, and the adsorbed mass would have shown a decrease from that of the pure EGCG solution in the presence of significant competitive binding. The rise in mass for the crude extract when compared to pure EGCG can be attributed to binding of secondary green tea molecules to BSA. Previous studies have shown that ECG and EC bind primarily in subdomain IIA [31, 39] and EGC binds in the entrance of the principal binding site in subdomain IIIA [39, 40]. In addition, other tea flavonoids were also found to bind within hydrophobic pockets in subdomains IIA and IIIA [41, 42], suggesting that each principal binding site contains multiple areas at which a diversity of molecules might simultaneously bind. It has also been shown that the hydrophobic binding sites on BSA are large compared to adsorbate molecules [43], giving BSA the ability to independently and simultaneously adsorb multiple ligands within the same binding site without significant interference between the ligands [44, 45]. Considering the above, the comparison between green tea and pure EGCG adsorption to BSA is possible, and it can be said that BSA allows for other green tea constituents to bind without affecting adsorption mode. Wang et al. [23] studied the adsorption of EGCG to BSA using QCM-D and concluded that the BSA-EGCG interaction is better described by the Freundlich isotherm. This suggests that EGCG does not form a simple monolayer on BSA. However, the correlation coefficient for the Langmuir model was also high in the previous study, suggesting that Langmuir isotherm assumptions of specific binding and monolayer formation should not be completely excluded, and the adsorption of EGCG to BSA could be described as a complex adsorption phenomenon that is best described by aspects of both the Langmuir and Freundlich models. Chitpan et al. used a similar approach to study the binding of black tea thearubigins to BSA and concluded that the binding behavior is best described by the Langmuir isotherm [46].

Figure 4 shows the adsorption rates as a function of concentration for EGCG and green tea. Each data point represents a single experiment on a clean crystal. The rate is defined as the slope of the tangent line at the beginning of adsorption as shown in Figure 2. It can be seen that the adsorption rate increases with increased solution concentration. This proportional increase is a further evidence of the phenomenological match to the Langmuir model. According to Langmuir, the rate of adsorption is proportional to the principal driving force, concentration [47].

The results of Figure 4 do not show significant decrease of adsorption rates for the crude green tea solution when compared to the EGCG solution. This further supports the hypothesis that competitive binding is not a dominant effect. Although other factors may influence the pharmacokinetics of catechins *in vivo*, these results indicate that using green tea instead of pure EGCG will not lower adsorption rates of green tea catechins to serum albumin.

## 4. Conclusions

A quartz crystal microbalance was used to investigate the binding characteristics of the principal green tea catechins to bovine serum albumin. Binding was examined using solutions of pure EGCG along with solutions of a crude green tea extract containing, in addition to EGCG, other catechins, flavonoids, and phenolic acid molecules. Based on this experiment, the following principal conclusions can be drawn:

1. The adsorption behaviors from both EGCG and green tea solutions can be described by the Langmuir isotherm. This is indicated by the relatively high coefficients of determination when the Langmuir model is used to fit the adsorbed mass vs. solution concentration data. The good fit to the Langmuir model indicates a homogenous coverage and concentration-driven specific binding. Higher adsorption rates at higher concentration also support the phenomenological fit of the Langmuir isotherm.
2. The equilibrium adsorbed mass from the green tea solution was higher than that from the EGCG solution. Since EGCG is the largest catechin in the green tea extract, the increase in equilibrium binding mass indicates that additional constituents are showing significant binding affinity for BSA. Competitive binding is found to be a secondary effect in dictating the equilibrium adsorbed mass, which is consistent with the ability of BSA to bind to many ligands simultaneously without significant interactions between these ligands.
3. There is no significant difference between the rate of adsorption from green tea solutions and that from EGCG solutions, further supporting the conclusion that competitive binding is nondominant.
4. The QCM label-free technique may be applied to the study of protein binding of crude mixtures such as botanical extracts in addition to solutions of pure compounds.

## Figures and Tables

**Table 1:** Isotherms parameters for EGCG and green tea adsorption onto BSA.

		EGCG	Green tea
Langmuir parameters	Γ_max_	5.0	7.9
	*α*	2.3 × 10^5^	3.2 × 10^5^
	*R* ^2^	0.68	0.70

Freundlich parameters	*k*_*f*_	99.5	18.0
	*n*	3.1	10.3
	*R* ^2^	0.59	0.12

**Table 2:** EGCG analysis of variance for Lineweaver–Burk linearization model.

Source	Degrees of freedom	Adjusted sum of squares	Adjusted mean squares	*F*-value	*P* value
Regression	1	0.92	0.92	15.47	0.01
Concentration	1	0.92	0.92	15.47	0.01
Error	7	0.42	0.06		
Lack of Fit	6	0.41	0.07	6.97	**0.28**
Pure error	1	0.01	0.01		
*Total*	8	1.34			

**Table 3:** Green tea analysis of variance for Lineweaver–Burk linearization model.

Source	Degrees of freedom	Adjusted sum of squares	Adjusted mean squares	*F*-value	*P* value
Regression	1	0.084	0.084	14.07	0.01
Concentration	1	0.084	0.084	14.07	0.01
Error	6	0.036	0.006		
Lack of Fit	4	0.016	0.004	0.42	**0.79**
Pure error	2	0.020	0.010		
*Total*	7	0.120			

## Data Availability

The data used to support the findings of this study are available from the corresponding author upon request.
